# Aberrant Gamma-Band Oscillations in Mice with Vitamin D Deficiency: Implications on Schizophrenia and its Cognitive Symptoms

**DOI:** 10.3390/jpm12020318

**Published:** 2022-02-20

**Authors:** Seungyeong Yu, Mincheol Park, Jiseung Kang, Eunkyung Lee, Jieun Jung, Tae Kim

**Affiliations:** Department of Biomedical Science and Engineering, Gwangju Institute of Science and Technology, Gwangju 61005, Korea; osos5918@gm.gist.ac.kr (S.Y.); eyejor@gist.ac.kr (M.P.); wltmd1006@gist.ac.kr (J.K.); eunkyung9732@gmail.com (E.L.); jje90626@gist.ac.kr (J.J.)

**Keywords:** vitamin D deficiency, perineuronal nets, evoked gamma-band oscillations, spontaneous gamma-band oscillations, schizophrenia, chondroitinase ABC

## Abstract

Vitamin D plays an essential role in cognitive functions as well as regulating calcium homeostasis and the immune system. Many epidemiological studies have also shown the close relationship between vitamin D deficiency (VDD) and the risk of schizophrenia. Cortical gamma-band oscillations (GBO) are associated with cognitive functions, such as attention and memory. Patients with schizophrenia show abnormal GBO with increased spontaneous GBO and decreased evoked GBO. However, the direct effect of VDD on GBO remains unknown. Parvalbumin interneurons, which predominantly contribute to the generation of GBO, are surrounded by perineuronal nets (PNN). We sought to investigate the associations among VDD, PNN, and GBO. Here, we injected a viral vector (AAV5-DIO-ChR2-eYFP) into the basal forebrain stereotaxically and implanted electrodes for electroencephalogram (EEG). At baseline, the evoked and spontaneous EEG power at the gamma frequency band was measured in 4-month-old male PV-Cre mice. After six and twenty weeks of vitamin D deficient food administration, the power of GBO was measured in the VDD condition. Next, we injected the chondroitinase ABC (ChABC) enzyme into the frontal cortex to eliminate PNN. We found that the VDD group showed decreased power of both optogenetically- and auditory-evoked GBO, whereas the spontaneous GBO increased. Enzymatic digestion of PNN showed similar changes in GBO. Taken together, we suggest that VDD could result in decreased PNN and, consequently, increase the spontaneous GBO and decrease the evoked GBO, reminiscent of the aberrant GBO in schizophrenia. These results show that VDD might increase the risk of schizophrenia and aggravate the cognitive symptoms of schizophrenia.

## 1. Introduction

Gamma-band oscillations (GBO) are brain rhythms in the frequency range of 30–100 Hz, as measured by electroencephalogram (EEG), and typically around 40 Hz [1]. GBO have been associated with cognitive functions such as attention, perception, and memory [2,3,4,5,6]. In addition, abnormal GBO have been observed with cognitive disorders such as schizophrenia, Alzheimer’s disease, and Fragile X syndrome, suggesting a neurological relationship between GBO and cognitive operations [7,8,9]. Additionally, several studies have shown that the entrainment of GBO using sensory, visual, auditory, electrical, and optogenetic stimulation can improve cognitive functions [10]. Given the various features of GBO, the exact mechanism is poorly understood. However, it is well known that the parvalbumin (PV) interneuron has a critical role in generating GBO [11,12], and optogenetics can selectively control PV neurons to regulate cortical GBO [13,14].

Schizophrenia is a mental disorder that involves positive, negative, and cognitive symptoms [15]. Patients with schizophrenia show deficits in cognitive performance [16], and animal models of schizophrenia report similar results [17,18,19]. Alterations of GBO in schizophrenic patients have been suggested as a possible mechanism for their cognitive deficits [1,7,20]. Patients with schizophrenia show reduced power of GBO in response to auditory [21], visual [22], transcranial alternating current stimulation (tACS) [23], and task [24]. However, the spontaneous (resting) GBO power is stronger in schizophrenic patients compared with healthy controls [25,26]. These paradoxical features of schizophrenia have been recently explained by differentiating evoked-GBO from spontaneous-GBO. Narrow frequency GBO respond to sensory stimulations or tasks, and GBO may correlate with cognitive performance [27]. Mice with impaired function of PV neurons exhibited a broadband increase in spontaneous gamma power but a decrease in task-evoked gamma oscillations [17,28]. The reduced function of PV interneurons increases the activities of cortical pyramidal cells and increases the E/I balance [27].

One of the neurobiological pathomechanisms in schizophrenia is the reduced perineuronal nets (PNN), a component of the extracellular matrix, found mainly around PV-expressing inhibitory interneurons, responsible for their fast spiking in the gamma frequency range [29]. Many previous studies have linked PNN with cognitive functions such [1] as memory, aging, and neurological disorders [30,31,32]. In particular, post-mortem studies have shown reduced PNN in the prefrontal cortex of subjects with schizophrenia [33,34,35]. Schizophrenia animal models, such as ketamine-treated rats and DISC1-mutated mice, also display reduced PNN in the prefrontal cortex [19,36]. Degradation of PNN alone is enough to decrease the firing frequency of fast-spiking neurons and results in an increased E/I ratio [37,38]. Although several studies have measured brain oscillations after eliminating PNN, the results are inconsistent [39]. This discrepancy seems to be due to different stimuli (e.g., visual vs. tactile), mouse state (e.g., freely moving vs. anesthetized), and the kinds of GBO (e.g., spontaneous vs. evoked).

Vitamin $D_{3}$ (Vitamin D) is produced from 7-dehydrocholesterol in the skin under ultraviolet B radiation [40]. Vitamin D deficiency affects multiple brain processes, including cognitive operations, in both healthy people and patients with neurological disorders [41]. Particularly, low levels of vitamin D can be a risk factor for schizophrenia. The relative risk of schizophrenia shows a fitted sine function of the month of birth, and it has a negative correlation with vitamin D concentration [42,43]. Vitamin D deficiency may downregulate the integrity of PNN, resulting in cognitive dysfunctions by disrupting calcium processing and producing nitric oxides [41]. One study suggests that adult vitamin D deficiency reduces PNN and results in abnormal network connections in the mouse hippocampus [44]. However, the direct impact of vitamin D deficiency on GBO is unclear. Therefore, we hypothesized that vitamin D and PNN are necessary for normal GBO and related function in freely moving mice. We sought to investigate the impact of vitamin D deficiency and disrupted PNN on the generation of GBO and cognitive function.

## 2. Materials and Methods

### 2.1. Animal

We used PV-Cre mice (Stock No. 012358, Jackson Laboratory, Bar Harbor, ME, USA). The mice were housed in temperature and humidity-controlled clean racks. The environment was maintained at a temperature and humidity range of 21 °C to 23 °C and 50% to 60%, respectively. The mice were maintained with free access to food and water, under a 12 h light/dark cycle (light on from 7:00 AM to 7:00 PM). For the vitamin D deficient group, vitamin D deficient chow (Cat. No. TD. 89123, ENVIGO, Indianapolis, IA, USA) was provided for 20 weeks, while the normal diet group maintained their standard chow (RodFeed, DBL, Eumseong, Korea), manufactured based on NIH-41 Open Formula. The normal diet consisted of 20% crude protein, 4% crude fat, 62% carbohydrate, and 8 IU/g vitamin D_3_ and the vitamin D-deficient diet was similarly formulated except without vitamin D (16% crude protein, 10% fat, 59% carbohydrate, and 0 IU/g vitamin D_3_). Animal care and all the experiments were conducted in accordance with the ethical guidelines of the Institutional Animal Care and Use Committee (GIST-2021-093).

### 2.2. Stereotaxic Surgery

At the start of the surgical procedures, mice were deeply anesthetized with 4% isoflurane (USP) delivered by a precision vaporizer (Classic T3 Anesthetic Vaporizers; Cat. No. 72-6468INT, Harvard Apparatus, Cambridge, MA, USA) to anesthetizing chambers. Once the mice were fully anesthetized, they were mounted in a mouse stereotaxic instrument (Cat. No. 51730, Stoelting Co., Chicago, IL, USA). Ketoprofen was diluted to 1 mg/mL with sterile 0.9 % saline and administered at a dose of 5 mg/kg. After the rostral-caudal incision, the stereotaxic operations set the target coordinates. For unilateral virus injection, the AAV5-DIO-ChR2-eYFP vector was injected into the basal forebrain region of the left hemisphere: AP: 0.0, ML: +1.6, DV: −5.5 mm. The viral vector was loaded in a NanoFil 10 µL syringe (NANOFIL, World Precision Instruments, Sarasota, FL, USA). The loaded syringe was connected to a microsyringe pump (UltraMicroPump III, World Precision Instruments, Sarasota, FL, USA) and controller ($\mathrm{Micro}4^{\mathrm{TM}}$, World Precision Instruments, Sarasota, FL, USA). A 1 µL viral vector was injected at a 30 nL/min flow rate. The syringe was inserted and pulled slowly and left in the target place for an additional 10 min to allow virus diffusion and avoid backflow along the needle tract. After the injection, an optical fiber was inserted into the same location as the virus. An optical fiber (0.39 NA, 200 µm core; Cat. No. FT200EMT, Thorlabs, Newton, NJ, USA) was used to apply a ceramic ferrule (1.25 mm diameter, 6.4 mm long; Cat. No. CFLC230-10, Thorlabs, Newton, NJ, USA). To record the frontal and parietal cortex electroencephalogram, five holes were drilled: frontal (AP: 1.0, ML: 1.0 mm), parietal (AP: −3.5, ML: 1.0 mm), reference (AP: −5.3, ML: 0.0 mm), ground (AP: −2.0, ML: 1.5 mm), Anchor (AP: −2.0, ML: −1.5 mm). Once all the electrodes and optic fiber were in place, the skull surface was completely covered with dental acrylic powder (${\mathrm{Fastray}}^{\mathrm{TM}}$ Custom Tray and Acrylic Base Plate Material; Cat. No. 0921381, Keystone Industries, Gibbstown, NJ, USA). The EEG/EMG headmount (Cat. No. 8402, Pinnacle Technology Inc., Lawrence, KS, USA) was positioned on the dental cement and connected to the electrodes. After the surgical procedure, the mice were allowed to recover for at least two weeks. All of our results and figures were analyzed with the frontal EEG.

### 2.3. Experimental Design

**Figure 1:**
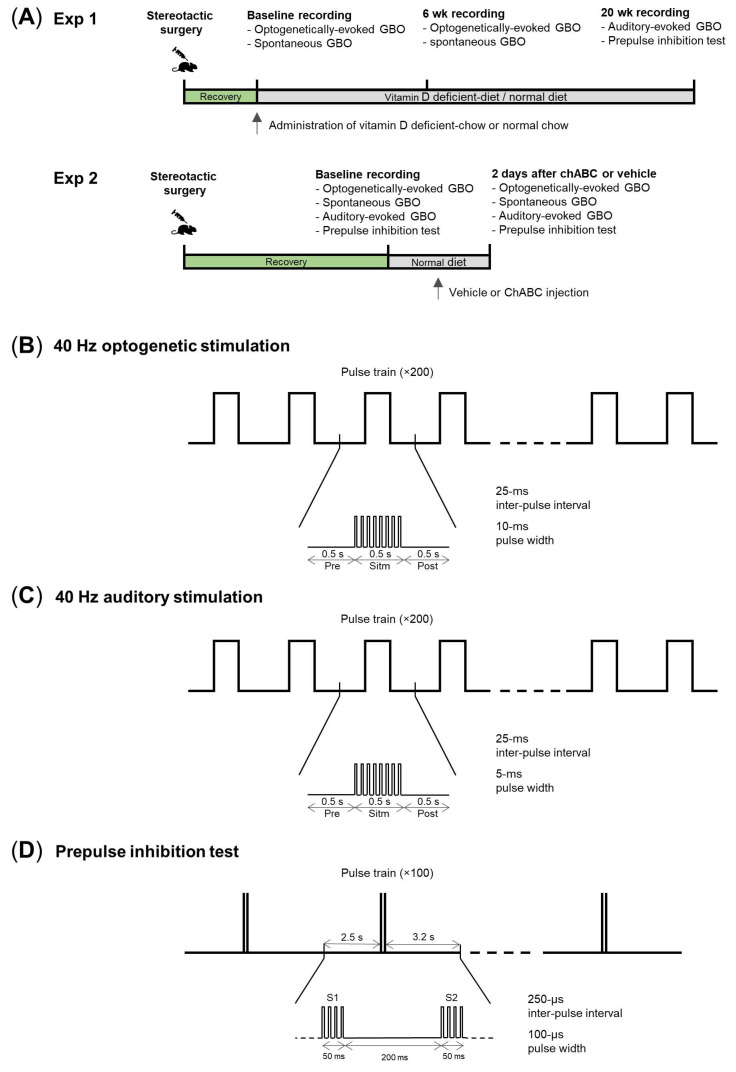
Scheme of the experimental schedules and the various methods for measuring frontal gamma-band oscillations (GBO). (**A**) Experimental timeline of the vitamin D-deficient diet group vs. normal diet group (VDD vs. ND, top) and the chondroitinase ABC-injected group vs. vehicle-injected group (ChABC vs. vehicle, bottom). (**B)** Stimulation protocol for 40 Hz optogenetically-evoked GBO. One trial was composed of pre-stimulation, stimulation, and post-stimulation (0.5 s each). During stimulation, a pulse occurs every 25 ms, and each pulse had a duration of 10 ms. A total of 200 trains were averaged and used for the power spectrum analysis. (**C**) Stimulation protocol for 40 Hz auditory-evoked GBO. The same protocol as for the optogenetically-evoked GBO was followed, except that each pulse had a duration of 5 ms. The sound was delivered with a sound pressure of 90 dB. (**D**) Stimulation protocol for the prepulse inhibition test. One train composed of two pulses (S1, S2) and 100 trains were averaged and used for power spectrum analysis. Each pulse continued for 50 ms, and a 5 kHz sound was delivered with 90 dB (6 s inter-trial interval, ITI; 250 ms inter-pulse interval, IPI).

We measured the frontal GBO in the normal diet and vitamin D-deficiency groups (ND and VDD, respectively, Figure 1A, Exp1). To measure optogenetically-evoked GBO, 4-month-old male PV-Cre mice were unilaterally injected with AAV5-DIO-ChR2-eYFP vector into the basal forebrain, according to the published methodology [13]. An optical fiber was also placed into the exact location of the vector, and cortical EEG electrodes were located on the frontal and parietal. After 2-week recovery period, we measured the baseline levels of the optogenetically-evoked GBO and spontaneous GBO. Furthermore, mice were separated into two groups and fed different food (VDD and ND). After 6-week from the baseline day, optogenetically-evoked GBO and spontaneous GBO were again measured in both groups. Finally, at 20 weeks from the baseline day, the auditory-evoked GBO and the prepulse inhibition test for the sensory gating experiment were measured. To directly manipulate the PNN, we set another experiment using the PNN digestion enzyme, ChABC (Figure 1A, Exp2). After 2-week recovery period, we measured the baseline levels of optogenetically-evoked GBO (Figure 1B), auditory-evoked GBO (Figure 1C), spontaneous GBO, and the prepulse inhibition test (Figure 1D). ChABC or vehicle was injected, and two days after the injection, all of the experiments were measured again, and the mice were sacrificed.

### 2.4. Optogenetically-Evoked GBO

Optogenetic stimulation was used to measure evoked EEG power in the gamma frequency band. The computer’s digital signal was converted into an analog signal for optogenetic stimulation, through a NI-DAQ (NI PCIe-6363; NI BNC-2110, National Instruments, Austin, TX, USA). To drive the laser light stimulation, a simultaneous input was provided to a 473 nm DPSS Laser (Cat. No. 21-01311-05, CNI Laser, Changchun, China). The laser and an optical fiber on the head of a mouse were connected through a mono fiber patch cord (MFP_2m_FC-ZF1.25; 0.22 NA, 200 µm core, Doric Lenses, Quebec, QC, Canada). Using WinWCP software, we made a stimulation protocol. To trigger a gamma signal at 40 Hz, a pulse occurs every 25 ms for 0.5 s, and each pulse has a duration of 10 ms. There were 0.5 s of rest time before and after the stimulation periods. Our protocol gave 200 trains using this 1.5 s train (0.5 s pre-stimulation, 0.5 s stimulation, 0.5 s post-stimulation).

### 2.5. Auditory Steady-State Response (ASSR)

Auditory stimulation for 40 Hz ASSR was used to measure sensory-evoked GBO. The computer’s digital signal was converted into an analog signal for auditory stimulation through a NI-DAQ, and the input was provided to the speaker (Cat. No. R50, Canston, Paju, Korea). Then, using WinWCP software, we made a stimulation protocol at 40 Hz. A pulse occurs every 25 ms for 0.5 s, and each pulse has a duration of 5 ms. There were 0.5 s of rest time before and after the stimulation periods. Our protocol gave 200 trains using this 1.5 s train, and the sound was delivered with a sound pressure of 90 dB in each chamber.

Two consecutive auditory stimulation at 5 kHz were used for the prepulse inhibition test. Using WinWCP software, we made a stimulation protocol at 5 kHz, 90 dB. A train was two pulses during 50 ms and consisted of a 250 ms inter-pulse interval (IPI), and a 6 s inter-trial interval (ITI). Our protocol gave 100 trains using these 6 s trains (2.5 s pre-stimulation, 50-ms first stimulation (S1), 50-ms second stimulation (S2), 3.2 s post-stimulation).

### 2.6. In Vivo Electrophysiology Data Acquisition

The mice were transferred to the recording chamber in a soundproof room to measure the evoked and spontaneous GBO. After a baseline recording, the mice were grouped into the ND and VDD groups. The VDD group was given vitamin D deficient chow, while the normal diet group sustained the normal chow. The preamplifiers (8200-K1-SL amplifier, Pinnacle Technology, Lawrence, KS, USA) were connected to the headmounts of the mice and the commutators. The EEG/EMG signal was amplified ×100, 100 Hz low-pass filtered, and sampled at 2 kHz, using the preamplifiers and the 8206 data conditioning acquisition systems (DCAS, Pinnacle Technology, Lawrence, KS, USA).

For all optogenetic stimulation experiments, the optical fiber on the head of the mice was also connected to a mono fiber patch cord. Using WinWCP software, the optogenetically-evoked EEG response was calculated by averaging the raw EEG records from all 200 trials for each mouse. An EEG power spectrum analysis was performed using Origin 2018b software. Spectrograms were generated for each mouse using short-time fast Fourier transform (STFT) (sampling frequency 2048; FFT length 4096; window length 128; overlap 64). Then power spectral density was calculated to generate the fold change of gamma at 40 Hz. The power segments at 40 ± 1 Hz during the pre-stimulation and stimulation periods were averaged to generate background and optogenetically-evoked EEG activity. The fold change in the gamma power means the ratio of the average gamma power during the stimulation period to the average gamma power during the time before the stimulation. For 40 Hz ASSR, an EEG power spectrum analysis was performed, as in the optogenetically-evoked 40 Hz experiment.

For the EEG power spectrum analysis in the prepulse inhibition test, the EEG response was calculated by averaging the raw EEG records from all 100 trials for each mouse. Spectrograms were generated for each mouse using STFT (sampling frequency 10002; FFT length 4096; window length 1024; overlap 512). The power segments at 40 ± 1 Hz during S1 and S2 were averaged to generate the gamma power of S1, S2, and the fold change.

Spontaneous GBO were measured using Sirenia Acquisition software (Pinnacle Technology, Lawrence, KS, USA). After three hours of habituation time, continuous EEG/EMG and synchronized video recordings were performed in freely moving mice (three hours recording from 10:00 PM to 1:00 AM). Vigilance states were scored manually for 10 s epochs, based on standard sleep scoring protocol, and used the wake epochs for the analysis. The wake epochs were manually screened based on low-amplitude mixed high-frequency EEG, high EMG, and movements in the video recording. The spontaneous GBO were the averaged power density of gamma frequency. The power density of gamma frequency is the gamma power of the EEG (35–45 Hz) divided by the full power of the EEG (0.5–1000 Hz). Delta (0.5–4 Hz), theta (5.5–8.5 Hz), alpha (8–13 Hz), and beta (13–30 Hz) were also measured.

### 2.7. Chondroitinase ABC Treatment

We prepared another experimental group to check the GBO before and after the enzymatic digestion of the PNN. Virus injection and optic fiber implantation were conducted, in the same way as in previous methods. We customized the guide cannula (C315GS-5/SP guide 38834 26GA, P1 Technologies, Roanoke, VA, USA) with an EEG wire to record local field potential (LFP). The customized cannula was implanted in the frontal cortex (AP: +2.0, ML: +0.5, DV: −0.5 mm), and a dummy cannula (C315DCS-5/SPC DUMMY, P1 Technologies, Roanoke, VA, USA) covered the hole of the guide cannula. The other four holes were drilled in the same positions as previously mentioned: parietal (AP: −3.5, ML: 1.0 mm), reference (AP: −5.3, ML: 0.0 mm), ground (AP: −2.0, ML: 1.5 mm), Anchor (AP: −2.0, ML: −1.5 mm). The EEG/EMG headmount (Cat. No. 8402, Pinnacle Technology, Lawrence, KS, USA) was positioned on the dental cement and connected to the electrodes. After the surgical procedure, the mice were allowed to recover for at least two weeks. All of our results and figures were analyzed with the frontal LFP.

We recorded the baseline data of the evoked GBO, spontaneous GBO, and the prepulse inhibition. Chondroitinase ABC (C3667-5UN, Sigma-Aldrich, Burlington, VT, USA) was dissolved in 0.1% BSA to a 50 U/mL concentration. The 0.1% BSA was made by mixing BSA powder (Probumin Bovine Serum Albumin, Millipore Corporation, Burlington, VT, USA) with 1× PBS. We injected the enzyme or vehicle using an internal cannula (C315IS-5/SPC INTERNAL 33GA, P1 Technologies, Roanoke, VA, USA) under isoflurane anesthesia. It was injected at 0.1 µL/min using a syringe (HSYR-1 SYRINGE 86200, Hamilton Company, Reno, NV, USA) connected to an internal cannula through a cannula tubing (C313CT/PKG TUBING. P1 Technologies, Roanoke, VA, USA). The GBO recordings were performed two days after the injection.

### 2.8. Immunohistochemistry

The mice were anesthetized with isoflurane and injected ketoprofen at a dose of 5 mg/kg. For perfusion and fixation, 1X PBS (Cat. No. P2007. Biosesang, Seongnam, Korea) and 4% PFA (Cat. No. PC00131-100-00, Biosesang, Seongnam, Korea) were used. The extracted brain was placed in a 15 mL conical tube containing ice-cold 4% PFA and kept in fixative overnight at 4 °C. The fixative brain was washed in 1× PBS three times at 120 rpm speed. After washing, the fixative brain was immersed in 30% sucrose solution (Cat. No. S0383-500G, Sigma-Aldrich, Burlington, VT, USA) at 4 °C, until the brain sank in the solution. The tissue was embedded in the OCT cryostat sectioning medium (FSC22 frozen section compound; Cat. No. 3801480, Leica Biosystems, Wetzlar, Germany) and stored at −80 °C until ready for sectioning. Using a microtome (Cat. No. SM2010R, Leica Biosystems, Wetzlar, Germany), the frozen brain was cut in 40 µm thick slices.

For quantification of PV and PNN, and the validation of the optic fiber, we stained coronal slices at AP: 2.0 and AP: 0 mm from the bregma. The slices were double-stained for both PV and PNN. To label PV, a sheep anti-parvalbumin (Cat. No. AF5058, R&D Systems, Minneapolis, MN, USA) was used at a dilution of 1:150 for the primary antibody. To label PNN, Biotinylated WFA (*Wisteria floribunda* agglutinin lectin) (Cat. No. B-1355, Vector Labs, Burlingame, CA, USA) was used at a dilution of 1:200. After overnight incubation of these two primary reagents, the tissues were washed with 1× PBS three times at 120 rpm speed, each for 5 min. As fluorochrome-conjugated secondary antibodies, we used streptavidin Alexa Fluor 647 (Cat. No. S32357, Invitrogen, Waltham, MA, USA) and donkey anti-sheep IgG Alexa Fluor 555 (Cat. No. A21436, Thermofisher Scientific, Waltham, MA, USA) at a dilution of 1:200 for 3 h at 80 rpm speed. After secondary antibody incubation, the tissues were washed with 1× PBS three times at 120 rpm speed. A confocal microscope (Cat. No. FV3000RS, Olympus, Shinjuku, Japan) was used for images. Paxinos and Franklin’s mouse brain atlas identified the mouse brain areas. The brains of the mice used for experiment 1 (VDD vs. ND) were imaged AP = 0, and the brains of the mice used for experiment 2 (ChABC vs. Vehicle) were imaged AP = 0, +2 mm. From the section of the brain, the cortical area was only used to calculate PV counts and the area of PNN, and the percent colocalization of PNN and PV among the PV neurons. Quantification was performed by ImageJ software (v1.53n).

### 2.9. Statistical Analysis

Statistical analyses of the experiments were performed using Prism 9 software. A Student’s *t*-test or Mann–Whitney U-test were used to compare unpaired conditions. A paired *t*-test or Wilcoxon signed-rank test were used to compare paired groups. An asterisk indicated *p* < 0.05, *p* < 0.01. Data were expressed as mean standard error.

## 3. Results

**Figure 2:**
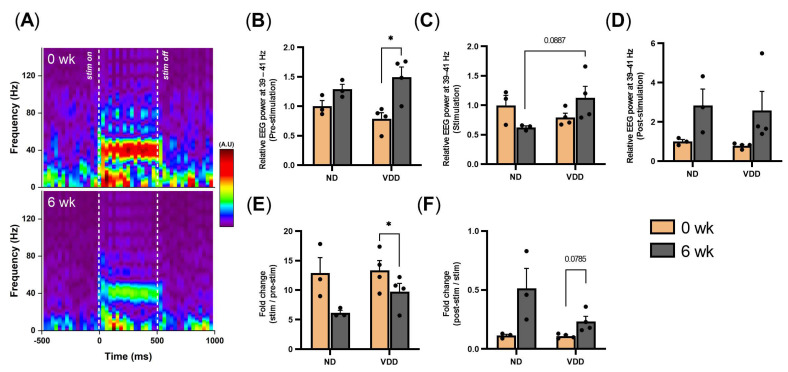
Optogenetically-evoked GBO reduced by vitamin D deficiency. (**A**) Representative time-frequency spectrogram of frontal electroencephalogram (EEG) from before and after vitamin D-deficient diet (0 wk, top; 6 wk, bottom). (**B**–**D**) Relative EEG power at 40 ± 1 Hz during the pre-stimulation, stimulation, and post-stimulation periods, respectively. (**E**) Fold change during the stimulation period in comparison with pre-stimulation period. (**F**) Fold change during the post-stimulation period in comparison with stimulation period. Data are presented as mean ± SEM. * *p* < 0.05 by the Student’s *t*-test.

First, we measured optogenetically-evoked GBO (40 ± 1 Hz) at the baseline and at six weeks from the beginning of the vitamin D deficient chow administration. The VDD group at six weeks showed a decreased fold change of GBO in comparison with the ND group (Figure 2A,E). To quantify the changes in detail, we also compared the power of GBO during each period, including pre-stimulation (−0.5 to 0 s), stimulation (0 to 0.5 s), and post-stimulation (0.5 to 1 s) periods (Figure 2B–D). Interestingly, GBO during optogenetic pre-stimulation increased more in the VDD than the ND group (Figure 2B), whereas that of stimulation and post-stimulation periods showed increasing trends without statistical significance (Figure 2C,D). In addition, the fold change of the power of post-stimulation compared to that of the stimulation period showed an increased trend in the VDD group (*p* = 0.079; Figure 2F).

**Figure 3:**
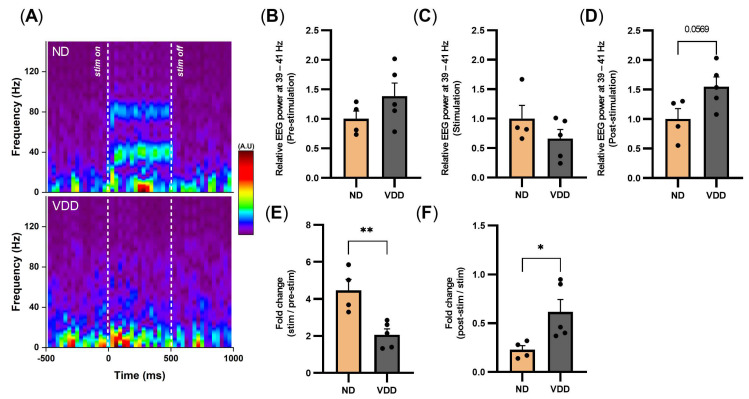
Auditory-evoked GBO reduced by vitamin D deficiency. (**A**) Representative time-frequency spectrogram of frontal electroencephalogram (EEG) from the normal and vitamin D-deficient diet at 20 weeks (normal diet, top; vitamin D-deficient diet, bottom). (**B**–**D**) Relative EEG power at 40 ± 1 Hz during the pre-stimulation, stimulation, and post-stimulation periods, respectively. (**E**) Fold change during the stimulation period in comparison with the pre-stimulation period. (**F**) Fold change during the post-stimulation period in comparison with the stimulation period. Data are presented as mean ± SEM. * *p* < 0.05, ** *p* < 0.01 by the Student’s *t*-test.

After 20 weeks of the vitamin D-deficient diet, we measured the auditory-evoked GBO between the VDD and ND groups using ASSR protocol. The 40 Hz auditory stimulation with a sound pressure of 90 dB could induce frontal GBO at 40 Hz, and this response was reduced in the VDD group compared to the ND group (Figure 3A). Although GBO during pre-stimulation, stimulation, and post-stimulation did not show significant changes (Figure 3B–D), the fold change during stimulation was significantly reduced in the VDD group (Figure 3E), and that of post-stimulation was significantly increased in the VDD group (Figure 3F).

**Figure 4:**
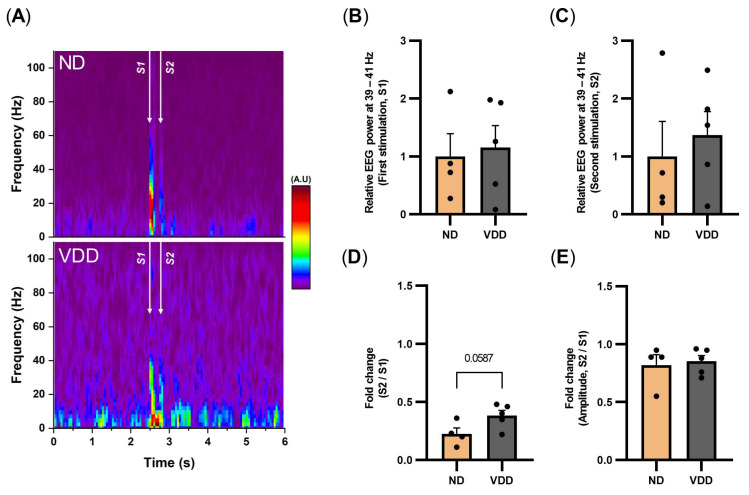
Prepulse inhibition reduced by vitamin D deficiency. (**A**) Representative averaged time-frequency spectrogram of frontal EEG from the normal diet and after the vitamin D-deficient diet at 20 weeks (ND, top; VDD, bottom). The arrows denote 5 kHz stimulation for 50 ms and the sound pressure was 90 dB. (**B**–**C**) Relative EEG power at 40 ± 1 Hz of stimulation 1 (S1, 2.5–2.55 s) and stimulation 2 (S2, 2.75–2.8 s). (**D**) Fold change during S2 period in comparison with S1 period. (**E**) Amplitude means the averaged traces’ peak amplitude during the stimulation period. Fold change during S2 period in comparison with S1 period. Data are expressed as mean ± SEM.

To assess the functional changes in the VDD group, we measured the sensory gating function using the prepulse inhibition paradigm at 20 weeks. In brief, we applied two consecutive acoustic stimuli and calculated the ratio of EEG responses (S2:S1; Figure 4A–D). The VDD group showed a trend of a higher S2:S1 ratio than the ND group (*p* = 0.059; Figure 4D), whereas there were no group differences in EEG responses to S1 and S2 (Figure 4B,C). The fold change in the peaks of the averaged EEG trace induced by S2 and S1 did not differ between the VDD and ND groups (Figure 4E).

**Figure 5:**
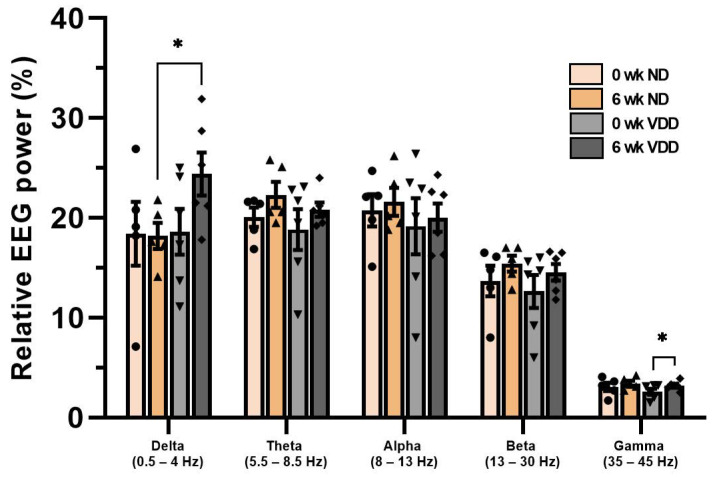
Spontaneous GBO increased by vitamin D deficiency. Frontal brain oscillations were analyzed by separating them into Alpha (8–13 Hz), Beta (13–30 Hz), Gamma (35–45 Hz), Delta (0.5–4 Hz), and Theta (5.5–8.5 Hz). Each frequency range’s data are normalized by total power (0.5–1000 Hz). Data are expressed as mean ± SEM, and *p < 0.05 by the Student’s *t*-test.

Next, we calculated the EEG power spectrum of the spontaneous EEG at baseline and at six weeks. From 3 h of EEG recording data, during zeitgeber time 15 to 18, we selected 10 s epochs of wakefulness, according to standard sleep scoring protocol. EEG power was analyzed in terms of five frequency ranges: Delta (0.5–4 Hz), Theta (5.5–8.5 Hz), Alpha (8–13 Hz), Beta (13–30 Hz), and Gamma (35–45 Hz). The relative EEG power was compared between the baseline and six week VDD, and we found a significant increase at the gamma frequency band (Figure 5). It is noteworthy that the spontaneous GBO was increased in the VDD group, whereas optogenetically- and auditory-evoked GBO were enhanced.

**Figure 6:**
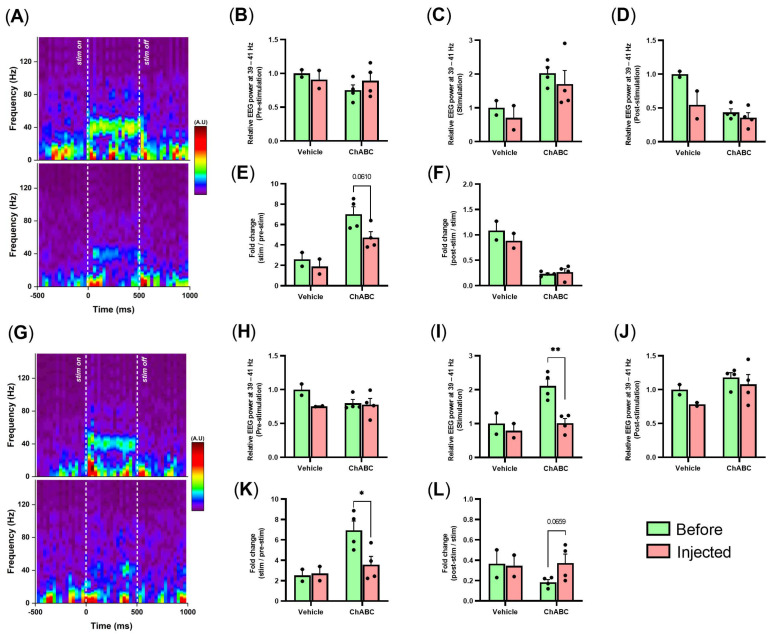
Optogenetically- and auditory-evoked GBO reduced by ChABC injection. (**A**) Representative optogenetically-evoked time-frequency spectrogram of frontal EEG from before and after ChABC injection (before, top; after, bottom). (**B**–**D**) Relative EEG power at 40 ± 1 Hz during the pre-stimulation, stimulation, and post-stimulation periods by optogenetic stimulation. (**E**) Fold change during the stimulation period in comparison with the pre-stimulation period by optogenetic stimulation. (**F**) Fold change during the post-stimulation period in comparison with the stimulation period by optogenetic stimulation. (**G**) Representative auditory-evoked time-frequency spectrogram of frontal EEG from before and after ChABC injection (before, top; after, bottom). (**H**–**J**) Relative EEG power at 40 ± 1 Hz during pre-stimulation, stimulation, and post-stimulation periods by auditory stimulation. (**K**) Fold change during the stimulation period in comparison with the pre-stimulation period by auditory stimulation. (**L**) Fold change during the post-stimulation period in comparison with the stimulation period by auditory stimulation. Data are presented as mean ± SEM. * *p* < 0.05 and ** *p* < 0.001 by the Student’s *t*-test.

**Figure 7:**
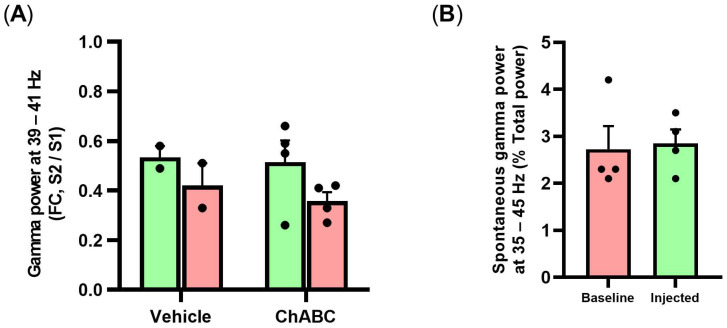
Prepulse inhibition test and spontaneous GBO in frontal cortex before and after ChABC injection. (**A**) Fold change during the S2 period in comparison with the S1 period. (**B**) Spontaneous GBO (35–45 Hz) was normalized by total power (0.5–1000 Hz). Data are expressed as mean ± SEM.

Based on the literature, we hypothesized that the reduction in PNN was the reason for the changes in GBO in the vitamin D-deficient state. Therefore, we conducted an additional experiment to eliminate PNN by applying ChABC, an enzyme to digest the cortical PNN, and measure the same parameters related to GBO. We found that the optogenetically-evoked GBO decreased in the ChABC-injected group (Figure 6A). In addition, the fold change of GBO was reduced in the ChABC-injected group (*p* = 0.0610; Figure 6E) while there were no differences in relative change (Figure 6B–D). Additionally, auditory-evoked GBO and the fold change of GBO decreased in the ChABC-injected group (Figure 6G,K). In auditory evoked-GBO showed powerful reduction in stimulation period and it was different from the increased power in pre-stimulation period of VDD (Figure 6H-J). Although the fold change of evoked GBO during post-stimulation period compared to the optogenetic stimulation period did not differ between before and after ChABC injection, that of auditory stimulation showed an increasing trend in the ChABC-injected group (*p* = 0.0659; Figure 6F,L). However, there were no changes in the prepulse inhibition test and spontaneous GBO, as well as the beta frequency band (Figure 7A,B, Supplementary Figure S1).

**Figure 8:**
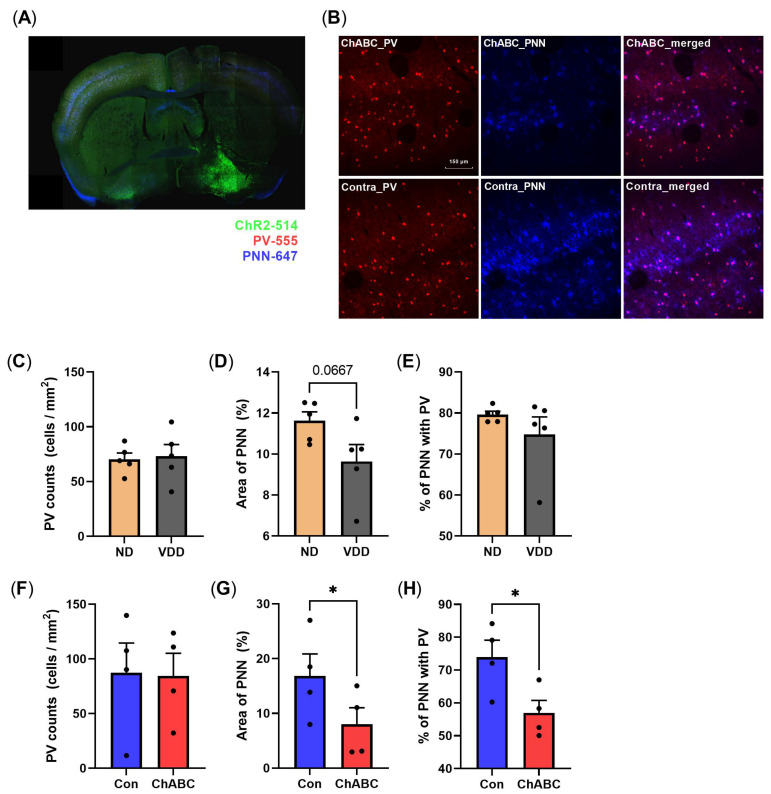
Immunohistochemistry for the quantification of PV and PNN. (**A**) Representative 10× confocal image for a whole-brain section of 20 week old vitamin D-deficient mouse. ChR2 expression in the left hemisphere basal forebrain (Green), parvalbumin (Red) and PNN (Blue) are expressed in the brain section. (**B**) Representative 30× confocal images of a ChABC-injected mouse. ChABC-injected left hemisphere (upper) showed a reduction of PNN compared to the contralateral hemisphere (lower). (**C**–**H**) The number of PV neurons, area of PNN, and colocalization percent of PV and PNN among all PV neurons. (**C**) PV counts between the normal diet group and vitamin D-deficient diet group. (**D**) Comparison of the area of PNN expression between the normal diet group and vitamin D-deficient diet group. (**E**) Percent colocalization of PNN and PV among all PV neurons between the normal diet and vitamin D-deficient groups. (**F**) PV counts between ipsilateral (ChABC) and contralateral (Con) sites of the ChABC-injected group. (**G**) Area of PNN expression between the ipsilateral and contralateral sites of the ChABC-injected group. (**H**) Percent colocalization of PNN and PV among all PV neurons between the ipsilateral and contralateral sites of the ChABC-injected group. Data are expressed as mean ± SEM. * *p* < 0.05 by the Student’s *t*-test.

Finally, we carried out immunohistochemistry to validate the site of the virus injection and optic fiber (Figure 8A), and quantified PV neurons and PNN in the VDD and ChABC injected groups (Figure 8C–E, Figure 8B, and Figure 8F–H, respectively). The ChABC-injected left hemisphere had a similar number of PV neurons, whereas the area of PNN was decreased compared to the contralateral side (representative histological findings; Figure 8B). The quantification of the immunohistochemistry showed a reduced area of PNN and percent colocalization of PNN and PV among all the PV neurons in the ChABC-injected group (both *p* < 0.05; Figure 8G,H), whereas the VDD group showed a decreasing trend of the PNN area compared to the ND group (*p* = 0.067; Figure 8C–E). 

## 4. Discussion

We found that VDD reduced optogenetically- and auditory-evoked GBO at around 40 Hz. However, the spontaneous GBO increased in VDD. In addition, the prepulse inhibition test revealed an impaired suppression of EEG response by the second stimulus. Next, we hypothesized that the underlying mechanism of these phenomena was a reduction of PNN in the cortex. Therefore, we repeated the quantification of GBO and the prepulse inhibition test in the PNN-disrupted condition. The enzymatic digestion of PNN using ChABC showed similar changes to the GBO shown in VDD, including reduced GBO by optogenetic and auditory stimulation. To the best knowledge of the authors, this is the first report to suggest VDD as a contributing factor to aberrant GBO. These results may indicate that VDD could be a risk factor exacerbating pre-existing abnormalities in GBO and sensory gating in psychiatric patients, such as those with schizophrenia.

GBO are one of the important biomarkers for cognitive dysfunction [7,8,9]. However, there are controversial results in GBO, partly because of the different experimental conditions used in measuring the power of GBO and/or the synchrony of GBO [39]. Hence, we measured GBO in a condition allowing natural behaviors, i.e., a freely moving condition, and applied multiple paradigms of quantification of GBO, i.e., evoked and spontaneous GBO. Specifically, we employed two different methodologies to evoke GBO using optogenetic stimulation of parvalbumin-positive neurons in the basal forebrain [13] and ASSR [21]. In addition, we also analyzed the spontaneous GBO without external triggers. We tried various approaches to GBO measurements and differentiated them from existing studies.

Reciprocal connections between PV interneurons and pyramidal neurons are critical in cortical GBO generation [11]. In particular, abnormalities in fast-spiking PV interneurons can promote aberrant GBO and cognitive dysfunction [14]. Furthermore, since PNN is known to underlie the fast-spiking of PV interneurons, the degradation of PNN has a direct effect on the aberrant GBO [37,38,39]. Therefore, studies showing that VDD reduces PNN suggest the relationship between VDD and GBO [44]. For example, VDD disrupts calcium processing and may reduce PNN due to pathomechanisms, such as increased nitric oxide secretion and elevated MMP-9 levels [41]. In our study, the pattern of reduced PNN and abnormal GBO were observed in VDD, and the abnormal GBO were reproduced in the treatment of PNN digestion. Taken together, our findings experimentally demonstrated that VDD could induce GBO abnormalities through PNN reduction.

The reduction of PNN due to VDD showed a decrease in optogenetically- and auditory-evoked GBO and an increase in spontaneous GBO. These different changes in evoked and spontaneous GBO could be due to the problem of the inhibitory process. In particular, decreased activities in PV interneurons due to PNN reduction leads to disinhibition of excitatory pyramidal neurons, which in turn can increase overall cortical activities [45]. The PNN disruption through ChABC treatment and knockout of brevican, a component of PNN, showed a decrease in the frequency of excitatory synaptic transmission to PV interneurons [46,47,48]. It can also cause disinhibition of excitatory pyramidal neurons and increased cortical excitability, reflecting the increase of spontaneous GBO [46,48]. In short, a reduction in the excitatory input to PV neurons and a decreased threshold for the generation of action potentials of PV neurons may result in decreased evoked and increased spontaneous oscillations, respectively. The increase in spontaneous GBO and the consequent decrease in signal-to-noise ratio can also cause functional abnormalities, such as reduced prepulse inhibition. It is well known that these abnormalities of GBO and prepulse inhibition appear in patients with schizophrenia [21,26,49].

Aberrant GBO have been studied in other neuropsychiatric disorders, such as Alzheimer’s disease, autism spectrum disorder, and mood disorder, as a common endophenotype of different clinical diagnoses. Patients with Alzheimer’s disease show increased or decreased GBO [8,50]. Patients with bipolar disorder or autism spectrum disorder show reduced GBO in response to 40 Hz ASSR [51,52]. Autism spectrum disorder in particular, also showed increased spontaneous GBO similar to our results [53]. We speculate that vitamin D deficiency could make an impact on the development or aggravation of clinical symptoms of some neuropsychiatric disorders. However, there is a paucity of studies for the link between vitamin D deficiency and GBO abnormality in such disorders. Investigation of the impact of vitamin D deficiency in the development and aggravation of psychosis, bipolar disorder, and other neuropsychiatric disorders is needed in the future.

Our hypothesis that VDD induces GBO abnormalities through decreased PNN was partially demonstrated by the direct manipulation of PNN using the injection of ChABC. In the ChABC treatment, optogenetically- and auditory-evoked GBO decreased, but spontaneous GBO and prepulse inhibition did not differ. Firstly, a crucial difference between diet-induced VDD and ChABC treatment lies in the fact that VDD affects globally and ChABC regionally. Although ChABC treatment has the advantage of direct digestion of the PNN, its impact should be limited to the area of injection because ChABC was injected locally. Therefore, the rest of the neural network responsible for generating GBO must be still intact and could have compensated for the negative effect of the local PNN reduction on generating spontaneous GBO and prepulse inhibition. On the other hand, because optogenetically- or auditory-evoked GBO involves a more specific neural network, such as the cortical projection of parvalbumin-positive neurons in the basal forebrain [13], and requires fast responses to the stimuli, a smaller area of PNN reduction, even in the ChABC treated mice, can result in a decreased level of GBO. Secondly, it should be noted that the impact of vitamin D deficiency is not limited to PNN reduction but may cause changes in gene expression by the genomic actions of vitamin D. For example, vitamin D can directly regulate the gene expression of parvalbumin [54]. This suggests that although the count of PV positive neurons remained unchanged in our study, the PV expression level may be decreased in vitamin D deficiency. Additionally, vitamin D downregulates mRNA expression for L-type voltage-gated calcium channels (L-VGCCs) [55]. Furthermore, L-VGCC can be attenuated by the reduction of extracellular matrix in relation to vitamin D deficiency [56,57]. Therefore, our ChABC treatment experiment could mimic only a phenomenon of PNN reduction among the various abnormalities vitamin D deficiency can cause.

We have several potential limitations in our experiments. The number of mice is not sufficient, making our results less conclusive by a trend-level significance. The timing of GBO measurements was 6 and 20 weeks in the VDD experiment and this difference potentially affected the results as a confounding factor. Because ChABC can reduce extracellular matrix non-specifically [58], the ChABC treatment should be interpreted with caution, although we found the decreased PNN in histology. In future experiments, analysis of GBO under the condition of vitamin D deficiency and MMP-9 inhibitor should be conducted to confirm the contribution of MMP-9 in aberrant GBO in vitamin D deficiency.

## 5. Conclusions

In our study, we used different methods to measure GBO, of which optogenetically- and auditory-evoked GBO decreased, and spontaneous GBO increased in the VDD group. The prepulse inhibition test showed the deficit of sensory gating function in VDD. These changes of GBO in VDD might be the influence of the loss of PNN. Enzymatic digestion of PNN showed similar results with VDD in optogenetically- and auditory-evoked GBO. In summary, vitamin D deficiency reduces PNN and causes schizophrenia-like GBO: decreased evoked GBO, increased spontaneous GBO, and sensory gating deficit. These results suggest that VDD might increase the risk of schizophrenia and aggravate the cognitive symptoms of schizophrenia.

## Data Availability

Data are available upon reasonable request to the corresponding author.

## Supplementary Materials

The following supporting information can be downloaded at: https://www.mdpi.com/article/10.3390/jpm12020318/s1, Figure S1: Spontaneous brain oscillations before and after ChABC injection (Delta, Theta, Alpha, Beta).
