# Supplementary material for: Aberrant Gamma-Band Oscillations in Mice with Vitamin D Deficiency: Implications on Schizophrenia and its Cognitive Symptoms
Source: J Pers Med. 2022 Feb 20;12(2):318. doi: 10.3390/jpm12020318 (PMC8879176; doi:10.3390/jpm12020318)
Supplement: Supplementary file 1 [file jpm-12-00318-s001.zip › jpm-1581385-supplementary.pdf]

Supplementary information

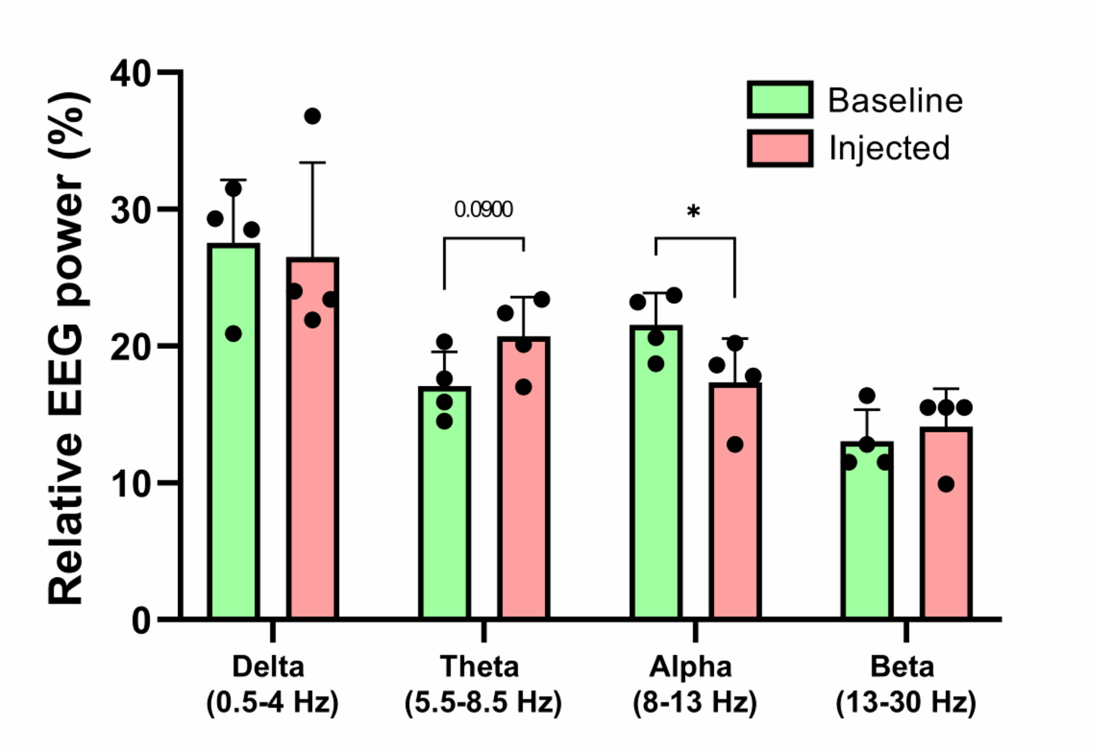

Supplementary Figure S1. Spontaneous brain oscillations before and after ChABC injection (Delta, Theta, Alpha, Beta). Gamma band oscillations (GBO) was shown in figure 7B. Data are expressed as mean ± s.e.m, and *$p < 0.05$ by Student's t-test.
